# Delineating bacterial community structure of polluted soil samples collected from cancer prone belt of Punjab, India

**DOI:** 10.1007/s13205-014-0270-5

**Published:** 2015-01-07

**Authors:** Gagandeep Kaur, Rohit Sharma, Kashmir Singh, Pushpender K. Sharma

**Affiliations:** 1 Department of Biotechnology, Sri Guru Granth Sahib World University, Fatehgarh Sahib, Pb India; 2 NABI, Mohali, Pb India; 3 Department of Biotechnology, Panjab University, Chandigarh, India

**Keywords:** 16S rRNA gene, Microbial diversity, Molecular phylogeny, Soil, Bioremediation

## Abstract

16S rRNA gene analysis has emerged as one of the valuable tools that are being utilized in investigating the molecular phylogenetic structure of the particular environment. Here, we embarked upon understanding and delineating the molecular phylogeny structure of microbes in polluted soil samples from cancer prone belt of the Punjab, India, which is highly contaminated with herbicide, pesticide and heavy metals. To investigate the bacterial phylogeny structure, a high-molecular weight metagenomic DNA was extracted from the soil samples, followed by PCR amplification, cloning and analysis of the 16S rRNA genes. Study employing 16S rRNA profiling of the community DNA revealed the presence of two major phylums: the *Proteobacteria* (26.7 %), the *Bacteroidetes* (11.2 %), and several minor groups, i.e., *Acidobacteria* (4.2 %), *Actinobacteria* (4.2 %), *Firmicutes* (2.8 %), *Verrucomicrobia* (2.8 %), *Gemmatimonadetes* (1.4 %) and *Chloroflexi* (1.4 %). Among the *Proteobacteria*, we mainly observed the *α*-*Proteobacteria* (18.3 %). Nearly, 38 % of the recovered 16S rRNA gene sequences in this study do not share similarity with known culturable bacterial sequences reported in the genebank data base and hence considered to be novel. More interestingly, 16S rRNA gene sequences of *archaeal* origin (7.0 %) were also recovered that primarily indicate change in their evolution pattern. A phylogenetic tree constructed based on alignment-dependent method revealed the extent of similarity these clones shared with each other, followed by alignment-independent methods that statistically confirmed the sequence variation among the clones. Despite the high level of contamination in the study area, we observed remarkable microbial diversity that mainly includes the Gram-negative bacteria. The presence of more Gram-negative bacteria indicates that they have evolved a robust mechanism to resist and cope up with these pollutants compared to Gram-positive groups. Investigation of the polluted soil samples employing culture-independent approach revealed important bacterial groups which could be engineered for future bioremediation studies.

## Introduction

Soil is a superabundant yet under-characterized ecosystem that represents an intricate and inexhaustible source of microbial diversity. The complexity of microbial diversity in soil is accredited to diverse type of interacting parameters, such as pH, water content, soil structure, climatic variations and biotic activity. The astonishing diversity of microbes present in the soil has immense potential in ameliorating our understanding about the soil microbial ecology. Furthermore, soil environment accommodates microbial species that can be a goldmine for the genes involved in biotechnological applications, such as biodegradation of man-made pollutants (Alexander 1977; Daniel 2005; Diaz 2004). Hence, exploring the diversity of versatile soil microorganisms is of paramount importance foreseeing their applications in numerous disciplines of biology. However, most of the soil microorganisms are exceptionally well adapted to their environment, and cannot be cultured under the usual laboratory settings. Current estimate connotes that more than 99 % of microorganisms present in their innate environment are not readily cultivable and, therefore, not accessible to applied and basic biotechnology research (Amann et al. 1995; Hugenholtz and Pace 1996; Ward et al. 1990). And hence, new and improved technologies are required to dissect the functional and structural diversity of the microbial species. Culture-independent approaches can circumvent such pitfalls and permit access to the genomes from entire communities that extend our comprehension about the diversity, ecology, evolution and functioning of the microbial world (Amann et al. 1995; Handelsman et al. 1998; Hugenholtz and Pace 1996; Riesenfeld et al. 2004; Ward et al. 1990).

The farmers in Punjab, India, are unseeingly using pesticides and chemical fertilizers to avert crop destruction, the extremity of these chemicals has resulted in extensive pollution of soil and water of Malwa region of the Punjab, due to which this region is experiencing the perilous repercussions, and has resulted in cancer and other dreadful diseases (Blaurock-Busch et al. 2014; Mittal et al. 2014). In the present investigation, we were aimed at studying the molecular phylogeny structure of the polluted soil samples of cancer prone belt of the Punjab, India, by employing 16S-rRNA gene analysis; this technique holds a great potential in characterizing the microbial diversity of contaminated environments (Nogales et al. 2001). Previously, several studies have been conducted to discern the phylogeny structure of several environment niches (Borneman et al. 1996; Ellis et al. 2003; Kuske et al. 1997; Quirino et al. 2009); however, to best of our knowledge, no studies have been undertaken to reveal the phylogeny structure of this environment. Therefore by analyzing the molecular phylogeny structure of this region, we wish to shed light into predominant microflora associated with such soil samples, and we strongly believe that it will enable researchers in designing better strategy to engineer these microorganisms and their metabolically active genes to combat pollution in such environment.

## Materials and methods

### Study areas and soil collection

The Punjab state is classified into three regions: Majha, Malwa and Doaba. Our study area is the Malwa region, which is south to river Satluj approximately between 29°30′ and 31°10′ north latitudes and 73°50′ and 76°50′ east longitudes. The study area comprises the following districts namely Fazilka, Bathinda, Mansa, Moga, Faridkot, Patiala, Sangrur, Barnala, Ferozepur, Muktsar and Ludhiana comprising an area of approximately 32,808 km^2^ (Mittal et al. 2014). Temperature in Malwa region ranges from 0 °C in winters to 47 °C in summers. The sites for the collection of soil samples were chosen on the basis of cancer prevalence in a particular area. We selected six agricultural fields from the different high cancer prone areas of the Malwa region. Samples were collected by the end of January and temperature at the time of sampling was 12 °C. Samples were collected in fresh autoclaved falcon tubes and were kept on dry ice during transportation from collection site to the laboratory, and were stored at −20 °C till use.

### DNA extraction and purification

Metagenomic DNA was directly extracted from 0.5 g of all the six soil samples employing XcelGen Soil gDNA isolation Kit (Xcelris genomics), as per manufacturer’s instructions. The extracted DNA was brown in color and was further purified according to the method of Sharma et al. (2007), using Q-Sepharose (Sigma). Q-Sepharose was washed and equilibrated with 10 mM potassium phosphate buffer (pH 7.2). Finally, approximately, 500 µl of solid washed beads were suspended in 1.5 ml of 10 mM potassium phosphate buffer. After mixing thoroughly, the aliquots of 300 µl of Q-Sepharose in buffer were transferred to six eppendorf tubes (1.5 ml) and centrifuged for 1 min to separate the overlaying buffer. Chromosomal DNA from the six soil samples was added into the centrifuge tubes containing Q-Sepharose beads, and mixed appropriately via inverting the tubes up and down gradually for 15 min. Tubes were kept at room temperature for 5 min. The Humic acids and polyphenolic compounds in the chromosomal preparation bound immediately to the Q-Sepharose. The preparations were centrifuged at 1,000×*g* for 1 min. Supernatant containing DNA was saved for further molecular manipulations.

### PCR amplification and cloning of 16 S rRNA

Purified DNA from all the six soil samples was used as a template for PCR amplification. The pair of primer used for the amplification was 342F (5′-CTACGGGGGGCAGCAG-3′) and 806R (5′-GGACTACCGGGGTATCT-3′) (Mori et al. 2014) and 27F (5′-AGAGTTTGATCMTGGCTCAG-3′) and 27R (5′-CGGYTACCTTGTTACGAC-3′) as potential forward and reverse universal primers, respectively. To amplify the 16 S rRNA genes, a touchdown PCR was performed in a Thermocycler (Veriti, Applied Biosystems) with the following thermal cycling conditions, 94 °C, 4 min, followed by 16 cycles of 94 °C for 50 s, annealing temperature was step-downs every cycle to 0.3 °C (from 55 to 50.2 °C) followed by extension at 72 °C for 2 min. The final PCR product was further amplified for another 15 cycles, at an annealing temperature of 50 °C, whereas the denaturation and extension phases were same as mentioned previously. Each 25 µl PCR contained 1 µl (0.1 µg) of total soil DNA, 1 μl of each primer (100 µM), 25 mM dNTPs (Thermo Scientific), 1 U *Taq* DNA polymerase (Thermo Scientific) and 1 × *Taq* reaction buffer (Thermo Scientific). A negative control reaction was also performed (having no DNA template). PCR amplification products were run on a 1.5 % agarose gel stained with ethidium bromide and bands of approximately 500, 900 bp and 1.4 kbp were excised and DNA was purified from gel slices using the XcelGen DNA Gel/PCR Purification kit (Xcelris genomics). The gel-eluted PCR products from all the six soil samples were pooled and cloned into the pGEM-T easy vector (Promega, USA) as per manufacturer’s instruction. The plasmid DNA was extracted from the randomly selected positive clones (~1,000 random clones were used) employing Hi Yield™ Plasmid DNA Mini Kit (Real Genomics).

### Nucleotide sequencing

Nearly 150 randomly selected recombinant clones were sequenced for the presence of 16S rRNA fragments. The nucleotide sequencing of cloned 16S rRNA genes was performed in an automated DNA sequencer ABI 3730xl (Applied Biosystems). Plasmid DNA preparations were carried out on microtiter plates according to ABI protocol and was used as a template for PCR cycle sequencing (Eppendorf) using the Big DYE Terminator Cycle Sequencing (Applied Biosystems) according to manufacturer’s instructions employing a T7 forward and SP6 reverse primer. To confirm the identities of each nucleotide, clones were sequenced twice.

### Sequence analysis and construction of phylogenetic tree

The DNA sequences obtained after sequencing were annotated and pasted as word file, and were further searched for % homology against the gene database at NCBI. Reads were edited by the removal of chimeras using DECIPHER (Wright et al. 2012), low-quality sequences and the formation of contigs using the CAP3 software (Huang and Madan 1999). The partial contigs of the 16S rRNA gene were compared to the non-redundant database of sequences deposited at the NCBI using BLASTN (Altschul et al. 1990). Results were used to determine that sequences were in fact from 16S rRNA and to determine their degree of similarity to previously known sequences. Multiple sequence alignment (MSA) was carried out using Clustal W (Thompson et al. 1994) with default settings. Phylogenetic analyses were performed with the Mega programs version 6.06 (Tamura et al. 2013) using the maximum parsimony method (DNA-PARS) with 1,000 bootstrap replicates for the generation of phylogenetic trees. The tree was displayed as radial tree.

### AIBIMM (Alignment-independent bilinear multivariate modeling)

In addition to alignment-dependent analysis, a non-alignment-based analysis of microbial community was also carried out via alignment-independent bilinear multivariate modeling (AIBIMM) approach, as described previously (Rudi et al. 2006). The sequences were transformed into multimer frequencies (*n* = 6) by computer program PhyloMode (http://www.matforsk.no/web/sampro.nsf/downloadE/Microbialcommunity). The multimer frequency data were compressed using principal component analysis (PCA). The starting and end point in the sequences were corrected using the normalize option data in PhyloMode. The two first principal components (PCs) were subsequently used for generating pairwise Euclidean distances for phylogenetic tree construction.

### Accession numbers

The nucleotide sequences described in this study have been submitted to the GenBank database of the NCBI under the Accession Numbers KM260222–KM260292.

## Results and discussion

**Fig. 1:**
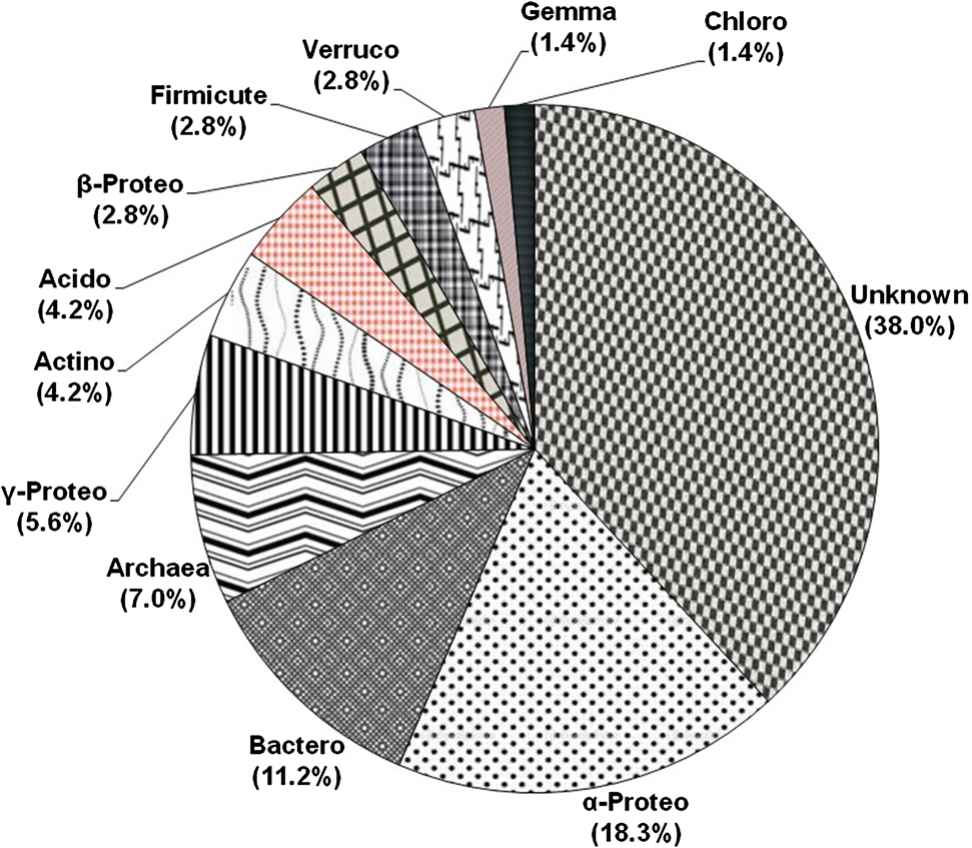
The percentage distribution of 16S rRNA genes. Each sector indicates percentage of clones within each phylogenetic group: unknown soil bacteria (unknown), *α-Proteobacteria* (α-Proteo), *Bacteroidetes* (Bactero), *Archaea*, *γ-Proteobacterium* (γ-Proteo), *Actinobacteria* (Actino), *Acidobacteria* (Acido), *β-Proteobacterium* (β-Proteo), *Firmicute, Verrucomicrobia* (Verruco), *Gemmatimonadetes* (Gemma), *Chloroflexi* (Chloro)

**Fig. 2:**
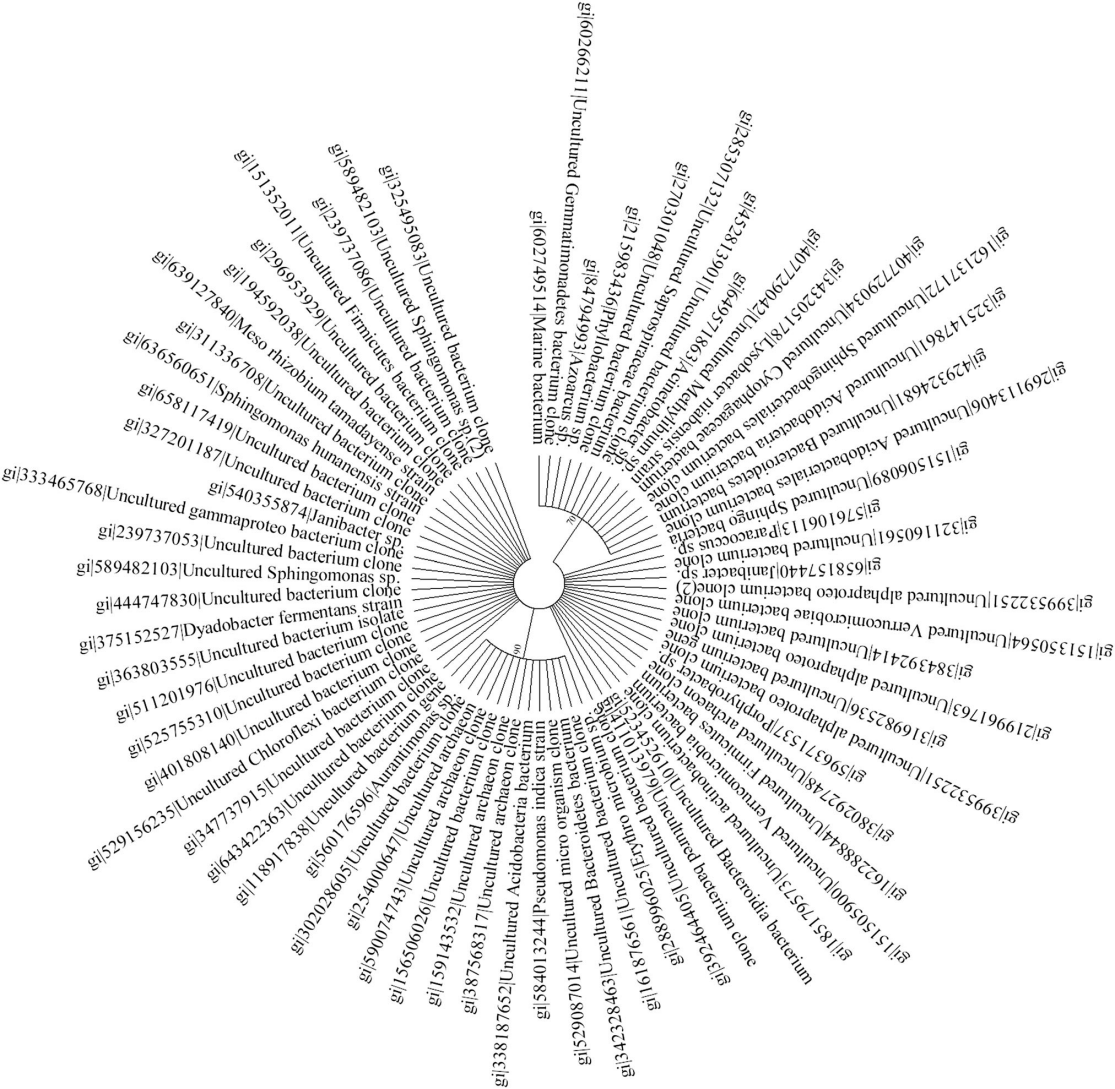
Phylogenetic tree of annotated sequences of 16S rRNA gene cloned directly from environmental DNA. The analysis involved 71 nucleotide sequences aligned with Clustal W (Thompson et al. 1994) and the evolutionary history was inferred by maximum parsimony method from MEGA6 programs (Tamura et al. 2013) using 1,000 bootstrap replicates. Unknown sequences are represented by a clone number

**Fig. 3:**
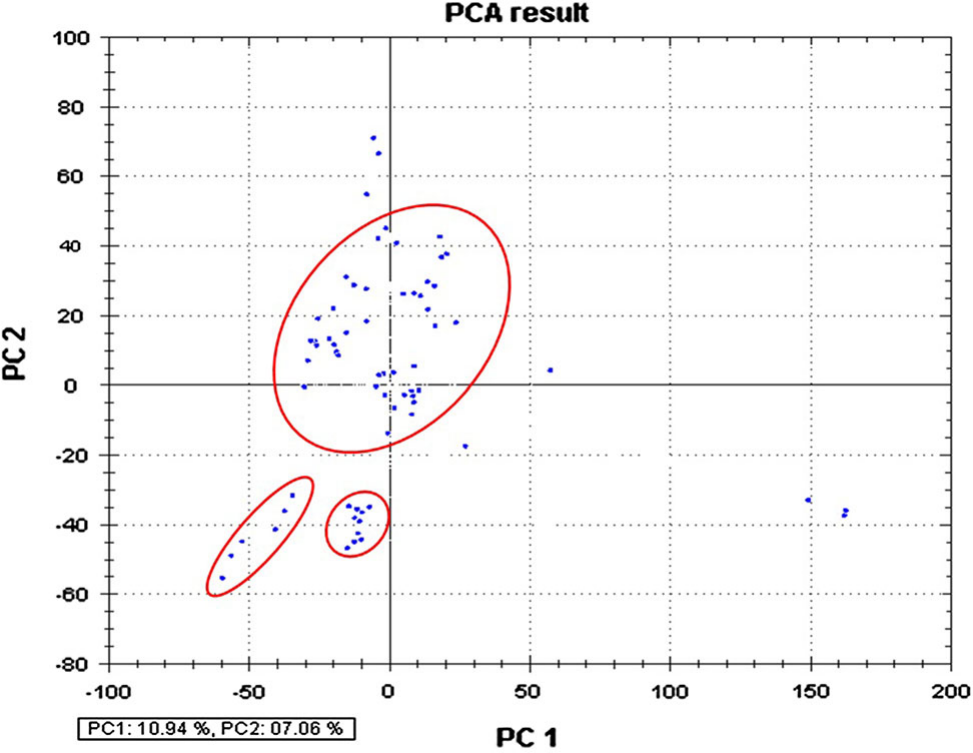
PCA analysis for soil bacterial community. The first principal component (PC1) is the line explaining 10.94 % variance. The second principal component (PC2) is the line that explains 07.06 % variance

The 16S rRNA gene analysis has become a valuable tool for studying the phylogenetic relatedness among microorganisms (Janssen 2006). Soil of Malwa region in Punjab (India) is known to be constantly contaminated with heavy metals and pesticides due to long-term agricultural practices in Punjab (Mittal et al. 2014; Blaurock-Busch et al. 2014). In our previous study, we also reported considerable presence of heavy metals and pesticides in the soil sample from this region (manuscript submitted in current science); therefore, we embarked upon studying the microbial diversity of the soil samples from this region to understand the distinctive ecosystem and the role of these microbes might play in maintaining ecosystem processes. To meet our aim, we extracted high-molecular weight metagenomic DNA corresponding to ~23 kb size that appeared to be brown in color, because of the presence of humic acids and polyphenolic compounds in it. Such contaminants are known to hinder molecular manipulations, i.e. PCR amplification and restriction enzyme digestion (Tebbe and Vahjen 1993). The DNA was, therefore, purified according to the method of Sharma et al. (2007) that resulted in pure colorless DNA (data not shown). Subsequently, the purified DNA was used for PCR amplification and cloning. Amplification of 16S rRNA genes from the environment DNA was carried out employing touchdown PCR that resulted in amplification of gene at three distinctive positions corresponding to ~500, 900 bp and 1.4 kbp. The PCR-amplified products were gel eluted and cloned into pGEM-T easy vector, thus a library representing ~1,000 clones was prepared. The plasmid DNA was extracted from the randomly selected clones, followed by sequencing of one-hundred and fifty recombinant clones. It is a well-studied fact that chimeras can result from the amplification of DNA directly extracted from an environmental sample (Wintzingerode et al. 1997). Among the 150 sequences analyzed, more than 40 appeared to be putative chimeric artifacts (vector sequences) which were detected using DECIPHER, and nearly 39 clones were of low-quality sequences, and hence were excluded from subsequent analysis. Finally, ~71 nucleotide sequences were analyzed for further studies. The recovered clones showed abundance according to group affiliation that included members of all the major phyla, commonly present in soil, as demonstrated in Fig. 1. The chimera-free and high-quality sequences were blasted in the GenBank (http://www.ncbi.nlm.nih.gov) using Blast N program. Based on similarity, the sequences were selected for the construction of the phylogenetic tree. To find out the phylogenic relatedness among the clones, we did both alignment-dependent and alignment-independent analysis. Phylogenetic tree was constructed based on MSA (multiple sequence alignment) and was analyzed via maximum parsimony. The tree was displayed as a radial tree (Fig. 2). In addition, a multivariate statistical approach was used to further explore the patterns in the sequencing data obtained. The prime objective behind using PCA was to gather the major information of the data and expressing it as a set of new orthogonal variables (principal components). The data gathered employing alignment-independent method provided a more comprehensive interpretation of the relationship among microorganisms. From the PCA plot analysis (Fig. 3), it becomes evident that there are 10.94 % variants in the PC1 and 7.06 % variants in the PC2. The pattern of points displayed in the plot clearly points towards the level of similarity between different groups of microorganisms which is further confirmed by the cluster formation. The points displayed outside the clusters distinctly reflect their difference from clustered microorganisms. All the clusters represent miscellaneous population of microbes. Interestingly, *Archaeal* population shows similarity with bacterial population. Phylogenetic tree (Fig. 2) demonstrated two major and several minor groups. Nearly 62 % sequences reported in the study shared 90–95 % similarity to cultivable bacterial groups; interestingly, 38 % sequences showed no homology to the cultivable bacterial groups and represent novel bacterial groups. A few (7.0 %) sequences of *archaeal* origin were also detected from this community that indicates change in evolution pattern of *archaea*. Previously, they were considered to be the domain of extreme environments. Previous studies have also documented their presence in non-extreme environments including marine (DeLong et al. 1994), terrestrial (Kudo et al. 1997), and in metal-contaminated ecosystems (Sandaa et al. 1999a). This implies that presence of *archaea* may have important ecological consequences in non-extreme habitats also. The majority of the bacteria discovered belong to two major phylums: the *Proteobacteria* (26.7 %) and the *Bacteroidetes* (11.2 %). Other minor groups were also observed that correspond to *Acidobacteria* (4.2 %), *Actinobacteria* (4.2 %), *Firmicutes* (2.8 %), *Verrucomicrobia* (2.8 %), *Gemmatimonadetes* (1.4 %) and *Chloroflexi* (1.4 %). Furthermore, several unidentified uncultured clones of novel bacterial origin (38.0 %) (Fig. 1) were also recovered. Among all the phylums identified, phylums *Actinobacteria* and *Firmicutes* constituted gram-positive bacteria (Rappe and Giovannoni 2003). From literature, it becomes evident that the phylums mainly found in soils are *Proteobacteria*, *Actinobacteria,* *Acidobacteria* and *Verrucomicrobia* (Janssen 2006). Analysis of the clones from bacterial origin revealed predominantly the presence of *Proteobacteria*, a phylum whose presence is well documented in contaminated sites (Ellis et al. 2003; Rastogi et al. 2010). Another major groups reported to be abundantly present in polluted soil are *Actinobacteria* and *Acidobacteria* (Briceno et al. 2012; Paul et al. 2006; Ward et al. 2009); however, we observed their insignificant presence in the present study. In contrast to this these species are reported to be abundantly present in the non-contaminated samples (Sandaa et al. 1999b; Sheik et al. 2012). Next, among α, β, γ, δ, ε subdivision of *Proteobacteria*, we mainly observed α-*Proteobacteria*, which is well correlated to previous studies conducted on metal-contaminated soil of Braunschweig, Germany (Sandaa et al. 1999b). In contrast, Joynt et al. (2006) identified δ *Proteobacteria* along with α, β, γ *Proteobacteria* in heavy metal-contaminated soil. The α-proteobacterial clones identified were of *Sphingomonas*, *Mesorhizobium tamadayense*, *Phyllobacterium*, *Porphyrobacter*, *Erythromicrobium*, *Paracoccus* and *Aurantimonas*. Under γ*-proteobacterium,* we observed sequence of *Lysobacter niabensis*, *Pseudomonas indica* and *Acinetobacter* origin. Among all, *Acinetobacter* is an important soil bacterium and has the potential to remove a wide range of pollutants (Abdel-El-Haleem 2003). Genus *Azoarcus* and *Methylibium* found in the study belong to *β-proteobacterium.* Additionally, the second most abundant phylum observed in this study was *Bacteroidetes* that harbor *Sphingobacteria*, *Saprospiraceae*, *Cytophagaceae*, *Dyadobacter fermentans*. Presence of *Bacteroidetes* has been previously reported in heavy metal-contaminated soils and sediments (Akob et al. 2008; Brodie et al. 2006; Ellis et al. 2003). The main feature of this bacterial community study is the abundant presence of uncultivable bacteria (38 %), and *Proteobacteria* which is one of the most abundant soil bacteria (Spain et al. 2009). Previous studies conducted on non-polluted environment samples have demonstrated variation in abundance of *Acidobacteria, Bacteroidetes, Firmicutes and Proteobacteria* (beta, gamma and delta) along with fewer phyla *Nitrospira, Deferribacteres, Chloroflexi*. The abundant presence of α-*proteobacteria* (18.2 %) in our study conducted on contaminated soil samples is well correlated to previous studies where they are shown to be present abundantly in contaminated soil, and scarcely reported in non-contaminated soil (Dhal et al. 2011; Sandaa et al. 1999b; Sheik et al. 2012). In addition to this Sandaa et al. (1999b), reported that the sequences from Gram-positive bacteria with a high DNA G+C content were more in soil with low metal inputs. These studies clearly demonstrate that microbial diversity strongly varies in two environments (contaminated and non-contaminated soil). Interestingly, identification of *Sphingomonas* from variety of contaminated environments is well documented, where they are implicated in degradation of numerous pollutants such as herbicides (Adkins 1999), Polycyclic Aromatic Hydrocarbons (PAHs) (Pinyakong et al. 2003), insecticides (Yu et al. 2013), heavy metals (Chien et al. 2008). This unique feature of the *Sphingomonas* bacterium may be attributed to molecular structure of its cell surface, which is reported to be more hydrophobic compared to other Gram-negative bacteria, due to the presence of glyco-sphingolipids instead of lipo-polysaccharides, hence has high ability to degrade many hydrophobic compounds or pollutants (Kawahara et al. 1999), and make it vital bacteria in bioremediation from various contaminated sites, which is also now becoming an intriguing area in research. Similarly, other bacteria belonging to *Alphaproteobacteria* also have appreciable biodegradative potential. The *Paracoccus* species are also implicated in biodegradation of various insecticides used in agricultural fields, such as chlorpyrifos as it can effectively degrade pyridine, a compound involved in the synthesis of chlorpyrifos (Lin et al. 2010; Xu et al. 2008) and fipronil (Kumar et al. 2012). Similarly, *Mezorhizobium* species are also implicated in chorpyrifos degradation (Jabeen et al. 2014). Furthermore, we did not observe *Deltaproteobacteria* in the present study, whereas they were reported to be quite abundant in the non-contaminated site (Dhal et al. 2011). The biodegradative capabilities of these bacterial consortia become more effective when they act together in association with each other (Fritsche and Hofrichter 2000) and culture-independent molecular approach enables us to identify such a huge diversity of microbial consortium which plays a major role in ecosystem processes. Despite high level of contamination in the study area, the remarkable microbial diversity observed stipulate versatility and adaptability of microorganisms to endure such environments. Altogether, investigation of the polluted soil samples employing culture-independent approach revealed important bacterial groups which could serve as a catalyst for future bioremediation studies. Our future goal is to sequence and characterize the metatranscriptome of the microbes present in the soil, and to provide functional insights into important metabolically active genes involved in degradation of these pollutants.
